# Rituximab Therapy for Double Seronegative Neuromyelitis Optica Spectrum Disease

**DOI:** 10.7759/cureus.60004

**Published:** 2024-05-09

**Authors:** Jose Carlos D Saballegue, Ma. Luisa Gwenn P Tiongson

**Affiliations:** 1 Center for Neurological Sciences, Quirino Memorial Medical Center, Quezon City, PHL; 2 Clinical Neurosciences, University of the East Ramon Magsaysay Medical Center, Quezon City, PHL

**Keywords:** double seronegative nmosd, neurophysiology, neurology, anti-mog, aquaporin 4, autoimmune, demyelinating, neuromyelitis optica spectrum disease, rituximab

## Abstract

Neuromyelitis optica spectrum disorder (NMOSD) is a rare central nervous system disease presenting as optic neuritis, myelitis, and brainstem syndromes. It may be aquaporin-4 seropositive, anti-myelin oligodendrocyte glycoprotein (MOG) antibody seropositive, or double seronegative. Double-seronegative NMOSD can pose a diagnostic and therapeutic challenge. Treatment typically aims to decrease the incidence of relapse, for which high-dose intravenous methylprednisolone is the first-line agent. Non-steroid treatments include azathioprine, mycophenolate mofetil, and rituximab. This case describes a 45-year-old female presenting with left arm numbness and weakness for three months. She had been previously diagnosed with optic neuritis in 2013 but was lost to follow-up. Progression of weakness warranted admission to the neurology department. Diagnostic work and imaging were suggestive of neuromyelitis optica. Tests for aquaporin-4, anti-MOG, immunoglobulin G, and immunoglobulin M in the cerebrospinal fluid were all negative. Initial treatment comprised methylprednisolone; however, due to the progression of symptoms, she was given two cycles of rituximab. Rituximab targets the CD20 antigen in B cells and is thought to reduce the risk of relapse and the severity of NMOSD. The patient’s Barthel index score, expanded disability status scale score, and motor examination improved after two cycles of rituximab.

## Introduction

Neuromyelitis optica spectrum disorder (NMOSD) is a rare central nervous system disease presenting as optic neuritis, myelitis, and brainstem syndromes. Although most patients with this disorder test positive for aquaporin-4 or myelin oligodendrocyte glycoprotein (MOG) antibodies, some are anti-MOG seropositive or double seronegative, which requires further investigation. Prevalence among white patients is 1 per 100,000 population, with an incidence of less than 1 per one million people annually. Among Asian patients, prevalence is 3.5 per 100,000 population. Clinical characteristics of NMOSD also differ according to race, with those of Asian or African descent having lower mean ages at onset [1]. In a comparison of seronegative and seropositive NMOSD cases, Kunadison et al. showed a 71% prevalence of aquaporin-4 seropositive in neuromyelitis optica and NMOSD, with more women in the seropositive group. They further suggested that aquaporin-4 antibody could be used as an inflammatory marker to evaluate disease severity and prognosis [2]. Approximately 18% of NMOSD cases are aquaporin-4 negative and anti-MOG negative.

Autoantibodies or autoimmune diseases are less common among those with aquaporin-4 negative NMOSD. Myelitis is common in the cervicothoracic area for those with double seronegative NMOSD, as indicated by magnetic resonance imaging (MRI) findings. Optic neuritis is the primary phenotype at onset in 30%-60% of cases of aquaporin-4 negative NMOSD and is usually bilateral, but it is less common in double seronegative NMOSD. However, long-term outcomes are less favorable in the double seronegative group [3]. A case report published by Alshurafa and Alkhateeb described a brainstem syndrome categorized under NMOSD but without optic neuritis and transverse myelitis [4]. Another case report by Khadka et al. involved a 35-year-old male presenting with weakness in the lower extremities diagnosed as double seronegative NMOSD. He developed optic neuritis and myelitis and was maintained on azathioprine and prednisone combination therapy [5].

Most (80% to 90%) patients will present as a relapsing episode of optic neuritis and myelitis. Relapse occurs in 60% of patients within one year and 90% of patients within three years, according to Wingerchuk et al. [6]. Neuromyelitis optica may also occur with concomitant systemic autoimmune diseases, such as systemic lupus erythematosus and Sjogren’s syndrome [6]. Anti-aquaporin-4 antibody is an established prognostic marker, and its presence indicates a high risk of relapses of optic neuritis and myelitis. Patients with NMOSD are more likely to have poor recovery, refractory pain, and permanent disability, and those with cervical affectation are at risk of respiratory failure [7]. As NMOSD is a potentially life-threatening condition that can lead to functional disability during acute attacks, treatment, which includes both steroid and non-steroid, aims to decrease the incidence of relapse [8]. High-dose intravenous (IV) methylprednisolone is the first-line agent to suppress NMOSD relapses. Non-steroid treatments include azathioprine, mycophenolate mofetil, and rituximab via intravenous route [9].

This case report involves a 45-year-old female with a three-month history of left arm numbness and weakness who had been diagnosed with optic neuritis in 2013 but was lost to follow-up.

## Case presentation

A 45-year-old female diagnosed with optic neuritis in her right eye in 2013 presented to the neurology emergency room with left arm numbness and a persistent pins and needles sensation throughout the day. She reported that the left arm weakness began two to three months prior to admission and progressed to numbness. She also experienced weakness progressing to numbness in her left leg, resulting in difficulty ambulating but no bowel or bladder incontinence. After consulting with a primary physician, she was prescribed vitamin B complex. The patient started experiencing blurred vision in the left eye, which prompted her to seek a consult with a neurologist within 24 hours. The neurologist advised her to be admitted to the neurology department.

**Figure 1:**
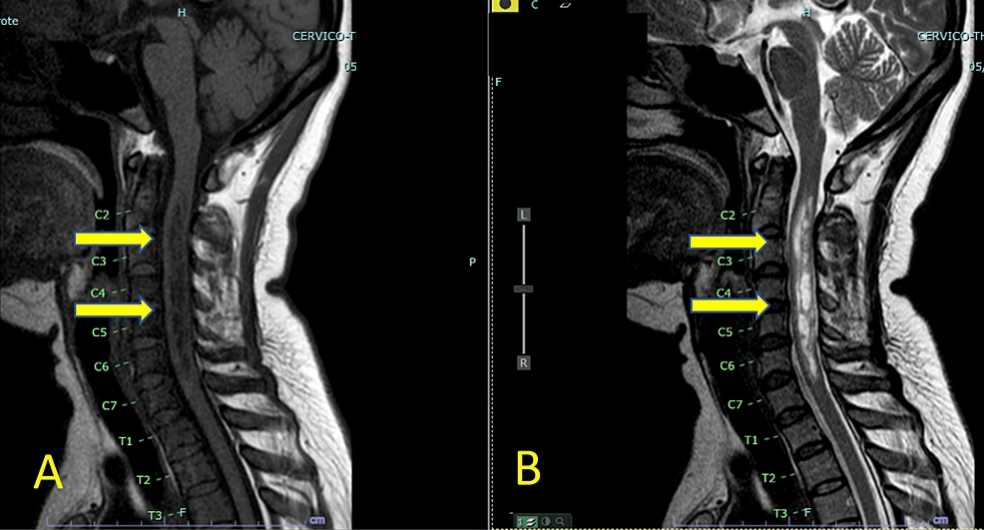
(A) T1 sequence showing longitudinal hypointensity with varying degrees of attenuation at the intramedullary area spanning from C2 to C7. No intra- or perilesional hyperintensity is observed. (B) T2 sequence showing varying degrees of hyperintensity at the intermedullary area spanning C2 to C7.

**Figure 2:**
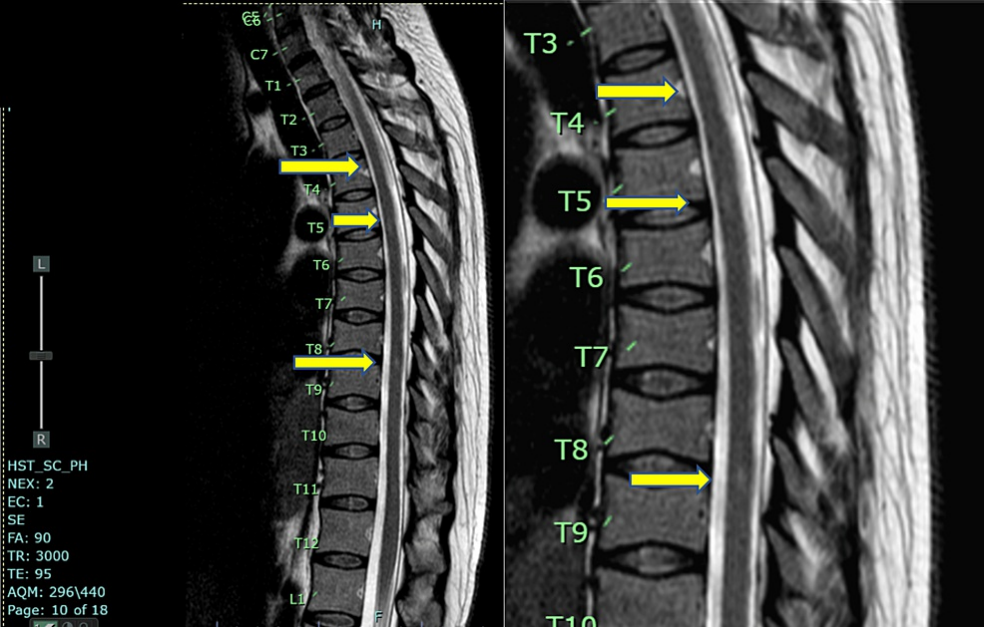
T2 sequence showing hyperintensities at T4, T8, T10, T11, and L1 vertebral bodies (arrows).

No other comorbidities were identified. Her family medical history included hypertension for both parents. Upon admission, her physical examination indicated her last normal sensory level was C6-C7 using all four modalities. Motor strength was graded at 5/5 for the right upper and right lower extremities and 3/5 for the left upper and left lower extremities. Visual acuity was 20/800 for the left eye, and the patient was able to count fingers in the right eye at a distance of 3 ft. The attending neurologist requested a cranial and spine MRI with contrast, which showed a longitudinal hypointense lesion with varying degrees of attenuation in the intramedullary area, spanning from C2 to C7 (see Figure 1). No intra- or perilesional hyperintensity was observed on the T2 sequence, but the lesion presented with varying degrees of hyperintensity in the intermedullary area spanning the same level. The same hyperintensity was seen at the T4, T8, T10, T11, and L1 vertebral bodies (see Figure 2).

A lumbar puncture was done, and the cerebrospinal fluid specimens tested negative for aquaporin-4, anti-MOG, oligoclonal bands, immunoglobulin G (IgG), and immunoglobulin M (IgM). She was diagnosed with double seronegative NMOSD and underwent treatment with IV methylprednisolone (1 g) once a day for five days. Her motor strength improved to 4/5 for the left upper extremities, and she was referred to rehabilitation medicine to start outpatient physical therapy.

**Figure 3:**
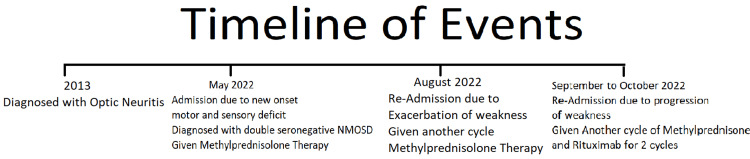
Timeline of events

The patient returned two weeks after discharge for exacerbation of weakness in the bilateral upper and lower extremities. Her motor strength was graded 4/5 for all extremities. A visual acuity test showed that she had no light perception in the right eye and a visual acuity of 20/50 in the left eye. The patient was readmitted to the neurology department, given another cycle of IV methylprednisolone (1 g) once a day for five days, and then discharged. One month after discharge, she was still experiencing progressive weakness in the left upper and lower extremities. A motor examination showed a motor strength of 4/5 in the right upper extremities, 3/5 in the right lower extremities, 1/5 in the left upper extremities, and 0/5 in the left lower extremities. Her visual acuity still showed no light perception in the right eye and 20/50 vision in the left eye. The patient was readmitted, and rituximab (1 g) and methylprednisolone (1 g) were administered intravenously for five days. After two weeks, the patient received a second cycle of rituximab. One week after treatment, the patient showed improvement in Barthel Index scores for bowel control and feeding. Her Expanded Disability Status Scale score was 8.5/10 after two cycles of rituximab. Her motor strength improved upon discharge to 4/5 for the bilateral upper extremities, 3/5 for the right lower extremity, and 2/5 for the left lower extremity. Visual acuity during discharge showed improvement in the right eye, as she was able to see hand movements; however, the left eye remained at 20/50. She continued outpatient physical rehabilitation after discharge. The timeline of events is given in Figure 3.

## Discussion

Our patient presented with optic neuritis and varying weakness in all extremities. The MRI result suggested neuromyelitis optica. After testing negative for both aquaporin-4 and anti-MOG antibodies, she was diagnosed with double seronegative NMOSD. Her symptoms initially responded to two cycles of methylprednisolone, but she was readmitted when symptoms progressed after discharge. We then administered another two cycles of rituximab. 

Rituximab is a chimeric monoclonal antibody made up of human IgG1 with variable regions from a murine anti-CD20 clone. CD20 is a marker of B cell lineage, and rituximab acts on the CD20 antigen on B cells. It is thought to reduce the risk of relapse and severity of neuromyelitis optica. The effect of rituximab on disability status and relapse incidence varies, but most had a reduction in relapse from 70% to 88% in 24 months in 2011. In 2013, 60% had been relapse-free for five years [9]. Rituximab induction treatment consists of either 1 g infused twice over a two-week interval or 375 g/m² once weekly for four weeks [8]. The most common adverse event reported during rituximab treatment is an infusion-related reaction, which occurs in 10.3% of cases [8].

Dysregulated B cell activity may cause neuromyelitis optica disease activity. Dysfunctional B cell tolerance may have a role in aquaporin-4 IgG production [9]. MRI findings have also suggested an absence of new or extending lesions after rituximab therapy, with minimal drug infusion-related adverse reactions. In our case, the patient was administered two cycles of rituximab (1 g) over two weeks, after which her Barthel Index score improved for bowel control and feeding. Other therapies being investigated for NMOSD include eculizumab, which targets the complement system, satralizumab, which targets the IL-6 receptor, and inebilizumab, which targets B cells [10].

## Conclusions

We describe the case of a 45-year-old patient with double seronegative NMOSD who was treated with methylprednisolone but still experienced progression of her symptoms. Seronegativity in this case was defined as testing negative for novel diagnostic immunologic markers such as aquaporin-4, CSF IgG and IgM, anti-MOG, and oligoclonal bands. As an autoimmune case, this implies that there are other immunologic markers yet to be discovered, which would further define or classify demyelinating diseases. This opens up opportunities for further research into yet-to-be-discovered immunologic markers that would not only define the disease but could also offer other treatments.

During the patient's hospital readmission, rituximab was administered, and the patient showed a good response. Rituximab therapy helped achieve remission and delay disease progression in this patient. Together with other therapies, such as methylprednisolone, rituximab could offer treatment for those with NMOSD. Other related drugs in development should be investigated for therapeutic benefits in patients dealing with this rare disease.
